# Algometer Precision for Quantifying Mechanical Nociceptive Threshold When Applied to the Udder of Lactating Dairy Cows

**DOI:** 10.3389/fvets.2018.00215

**Published:** 2018-09-12

**Authors:** Catarina Krug, Trevor J. Devries, Jean-Philippe Roy, Jocelyn Dubuc, Simon Dufour

**Affiliations:** ^1^Département de Pathologie et Microbiologie, Faculté de Médecine Vétérinaire, Université de Montréal, St-Hyacinthe, QC, Canada; ^2^Canadian Bovine Mastitis and Milk Quality Research Network, St-Hyacinthe, QC, Canada; ^3^Department of Animal Biosciences, University of Guelph, Guelph, ON, Canada; ^4^Département de Sciences Cliniques, Faculté de Médecine Vétérinaire, Université de Montréal, St-Hyacinthe, QC, Canada

**Keywords:** dairy cattle, udder, nociception, algometer, precision, reliability

## Abstract

Objectives of this study were to: (1) quantify the reliability of an algometer for measuring mechanical nociceptive thresholds when applied to the udder of dairy cows; and (2) evaluate whether covariates, such as cow characteristics or time of the day, would influence algometer measurements. This prospective study was performed in a university herd of 37 lactating cows during five consecutive days, involving two raters. Two types of measurement were obtained: one qualitative binary measure (i.e., reaction vs. no reaction) and one quantitative measure presented in kilograms (i.e., mechanical nociceptive threshold, MNT) for the cows that reacted. Kappa statistics were used to investigate test-retest and inter-rater reliability for the qualitative measure, while concordance correlation coefficient (CCC) and limits of agreement plot were used for the quantitative measure. Whether algometer measurements were influenced by several covariates (i.e., time of the day, level of milk production, days in milk, and parity) was then evaluated using logistic or linear regression models, depending on the outcome. The algometer was moderately reliable; there was moderate test-retest reliability (Kappa = 0.53; CCC = 0.58) and inter-rater reliability (Kappa = 0.42; CCC = 0.54). The MNT varied substantially as a function of time of the day and parity. This is the first study reporting reliability of a pressure algometer for quantifying MNT and investigating covariates possibly affecting this measurement when applied to the udder of dairy cows. It is concluded that the use of an algometer for quantifying MNT on the udder is only moderately repeatable and is influenced by extraneous covariates. Its usage in research setting to quantify changes in sensitivity at the udder level should, therefore, be considered very cautiously or it should be further developed.

## Introduction

Practices such as abrupt cessation of milking at drying-off (1), prolonged milking intervals (2), and incomplete milking at the beginning of the lactation (3, 4), may lead to udder distension, milk leakage, and inflammatory responses (5, 6). Under such conditions, the animals may experience increased mechanical sensitivity, perhaps to a level where their welfare is negatively affected (7).

Handheld pressure algometers have been used to assess sensitivity in dairy cattle in cases of mastitis (8), lameness (9), or after dehorning (10). The rater exerts pressure with the device in the body region of interest until the animal responds with an avoidance reaction (i.e., kicking, shifting weight). The mechanical nociceptive threshold (MNT) represents the amount of force (in kg) necessary to trigger animal avoidance response, measuring animals' sensitivity. Although algometers have been shown to be reliable in humans (11) and in some domestic animals [e.g., dogs: (12); horses: (13); piglets: (14)], formal evaluation and validation of handheld algometers for quantifying MNT on the udder of dairy cows appears to be lacking.

The aims of the current study were to: (1) quantify the reliability of algometer measurement for quantifying MNT when applied to the udder of dairy cows; and (2) evaluate whether extraneous covariates, such as time of the day or cow characteristics (parity, stage of lactation, or production level), influence algometer results. Specifically, we hypothesized that if algometer measurement was influenced by these covariates, then care should be taken in standardizing, if possible, for those variables when using pressure algometers.

## Materials and methods

This experiment was performed with the permission of the Animal Ethics Committee of the *Université de Montréal* under reference number Rech-1701. Observations from animals presenting clinical mastitis during the study were excluded from the analyses, because clinical mastitis may influence algometer measurements (8). Clinical mastitis was diagnosed by the farm manager and defined as presence of abnormal milk or typical inflammation signs (swelling, redness, pain) of the mammary gland (15). Only animals clinically healthy were selected for the study.

### Algometer measurements

**Figure 1:**
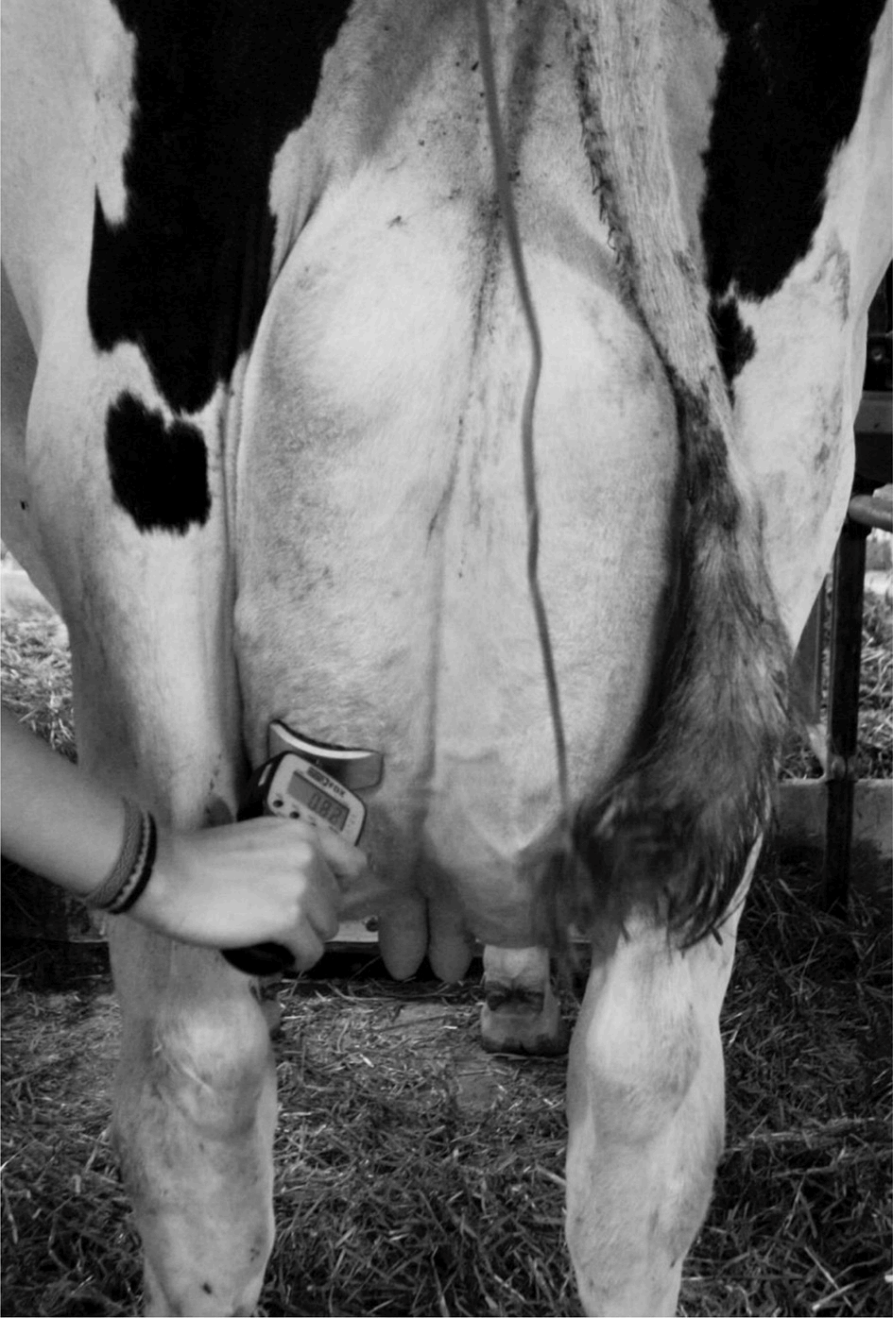
Illustration of the placement of a pressure algometer for quantifying mechanical nociceptive threshold in dairy cows.

An observational prospective study was conducted at the *Institut de Technologie Agroalimentaire* teaching farm (Saint-Hyacinthe, QC, Canada) from July 6 to 10, 2015. The teaching farm had 37 milking cows that were housed in a tie-stall facility, and produced a mean 305-d milk yield of 11,301 kg. Stalls' resting surface were 1.52 m wide by 1.80 m long and consisted of a rubber mat covered with a small amount of straw. Most cows had two neighbors, except four cows located at the very end of the two alleys. Milking occurred twice a day, at 07:00 h and 16:00 h. An algometer (Force Ten FDX 50; Wagner Instruments, Greenwich, CT) was used to measure the MNT. A concave probe head with 24.2 cm^2^ was added to the pressure point of the algometer to assure a good adaptation to the udder anatomy, and to avoid discomfort due to algometer surface (Figure 1). Before exerting pressure with the algometer, the rater would touch at the cow's upper hind leg, so that she would perceive the rater's presence. If lying, the cow would be encouraged to stand up for the measurement. Using the algometer device, the pressure would be exerted perpendicular to the skin of the inferior third of the left hind quarter, while the animal was standing, as shown on Figure 1. When the cow reacted by kicking or shifting weight, the quantitative measure on the algometer (in kg) was noted. When the cow did not react at the raters' maximum pressure, the lack of reaction would be noted, along with the pressure applied (for descriptive reasons), but this latter measure was not considered as the MNT. Therefore, two types of result were recorded, one qualitative binary measure (reaction vs. no reaction) and one quantitative measure (i.e., MNT, in kg) for the cows that reacted.

To investigate algometer reliability, two veterinarians used the device for measuring (twice each) the MNT of all individual cows immediately prior to the afternoon milking, for five consecutive days (Monday to Friday). Before farm sampling begun, the raters reviewed how to use a pressure algometer based on the methodology of Fitzpatrick et al. (8). Rater one always recorded before rater two, and all measurements were taken within a short time frame (i.e., 2–5 min). Raters were neither blinded to their own results, nor to the other rater's results.

To evaluate whether algometer measurement was affected by covariates, additional algometer measurements were collected by one of the raters five times a day: immediately after morning milking (time 0), + 4:00 h, + 5:30 h, + 7:30 h following morning milking, and immediately prior to afternoon milking (+ 9:00 h). Days in milk (DIM), parity, and milk production obtained in the afternoon milking of each of the 5 d of study were also recorded. Parity was categorized in 1, 2 or ≥3. Days in milk were categorized in early (0–100 DIM), mid (101–200 DIM), and late lactation (>200 DIM).

### Definition of terms

*Reliability* refers to the consistency of different observations/measurements of the same object by the same rater (test-retest reliability, or *repeatability*) or by different raters (inter-rater reliability, or *concordance*). When investigating tests for which the rater may influence the results, both inter-rater reliability and test-retest reliability should be assessed (16), as the lower the reliability is, the higher the measurement error. Measurement error includes both systematic error, or bias, and random error. *Precision* is a measure of variability due to random error, and therefore, it is related to reliability (17).

*Agreement* is distinguished from reliability, as the former corresponds to how close the results of the repeated measures are. Both reliability and agreement are important when assessing measurement properties of an instrument such as the algometer, since validity would be impaired if a measurement would not be adequately consistent (18).

Algometer *validity* relates to how close the MNT obtained with an algometer is to the actual level of udder sensitivity of a cow. The importance of any diagnostic technique, including an algometer, is judged in terms of its reliability and validity. Since there is no gold standard to measure udder sensitivity, direct assessment of validity is impossible. However, if a measurement is affected by other extraneous factors (i.e., factors not associated with what we want to measure), then the validity of the measurement can be questioned (17).

### Statistical analyses

#### Reliability

Kappa statistics, concordance correlation coefficients (CCC), and limits of agreement plots were used to determine test-retest reliability and inter-rater reliability (19). Kappa statistics were used to assess agreement of qualitative outcomes within and between raters. Kappa values under 0 were considered poor agreement, 0.01–0.20 slight, 0.21–0.40 fair, 0.41–0.60 moderate, 0.61–0.80 substantial, and 0.81–1.00 almost perfect agreement (19).

Reliability of the algometer's quantitative result was calculated using CCC and limits of agreement plots. The CCC measure a linear association between two measurements and classifies in several degrees of agreement (19): poor, < 0.20; fair, 0.21–0.40; moderate, 0.41–0.60; good, 0.61–0.80; and very good, 0.81–1.00. The limits of agreement plot presents graphically the difference against the mean between two measurements. The latter is especially useful in detecting patterns of disagreement between raters/measurements, consequently helping to understand the origin of discrepancies (19).

To further detail test-retest reliability and inter-rater reliability, the effect of raters (A or B) and of order of the four measurements (1st, 2nd, 3rd, or 4th) collected repeatedly on one time point on both the qualitative binary measure (reaction vs. no reaction) and on the MNT were investigated independently (i.e., in univariate models). Mixed logistic and linear regression models were used to investigate effect of these two covariates on the qualitative binary measure (reaction vs. no reaction) and on the MNT, respectively. Clustering of algometer measurements by day, and by cow (4 measurements/d/cow) was accounted for using random day and cow intercepts. The logistic mixed models were as follows:

(1) $$\begin{array}{l} {\text{Y}}_{\text{ijk}}\sim \text{bin}\left[\text{P}\left({\text{Y}}_{\text{ijk}}\right)\right]\\ \text{Logit}\left[\text{P}\left({\text{Y}}_{\text{ijk}}\right)\right]=\beta_{0\text{ijk}}+\beta_{1}{\text{X}}_{\text{ijk}}+{\text{v}}_{0\text{k}}+{\text{u}}_{0\text{jk}}+{\text{e}}_{0\text{ijk}} \end{array}$$

where Y_ijk_ was the reaction (or lack of reaction) at the i^th^ measurement of the j^th^ day of the k^th^ cow, which was a function of a predictor X (raters A or B; or sampling order, 1st, 2nd, 3rd, or 4th) through the logit function, and followed a binomial distribution with prevalence P of presentation of avoidance reaction. β_0_ was the intercept and β_1_ the regression coefficient for the effect of the predictor X. The cow, day, and measurement error terms, were represented as v_0k_, u_0jk_, and e_0ijk_, respectively.

The linear mixed models were as follows:

(2) $$\begin{array}{l} {\text{MNT}}_{\text{ijk}}\sim \text{N}\left(\mu ,\sigma \right)\\ {\text{MNT}}_{\text{ijk}}=\beta_{0\text{ijk}}+\beta_{1}{\text{X}}_{\text{ijk}}+{\text{v}}_{0\text{k}}+{\text{u}}_{0\text{jk}}+{\text{e}}_{0\text{ijk}} \end{array}$$

where MNT_ijk_ was the MNT (in kg; with mean μ and variance σ) for the i^th^ measurement of the j^th^ day from the k^th^ cow. β_0_ was the intercept and β_1_ was the regression coefficient for the predictor X (raters A or B; or sampling order, 1st, 2nd, 3rd, or 4th). v_0k_, u_0jk_, and e_0ijk_ were the cow, day, and measurement error terms, respectively, all assumed to follow an approximately normal distribution.

For the latter model, the fit of different covariance structures (compound symmetry, autoregressive, autoregressive moving average, Toeplitz, and heterogeneous variance compound symmetry) was compared using the Akaike Information Criterion. The compound symmetry correlation structure (equivalent to a conventional random intercept) was shown to provide a fit similar to that of more complex structures and was, therefore, retained for these analyses.

#### Relationship between algometer measure and covariates

Similarly to the previous section, logistic and linear regressions were used to investigate effect of covariates on the qualitative binary measure (reaction vs. no reaction) and on the MNT, respectively. Clustering of algometer measurements by day, and by cow (5 measurements/cow/5 d) was accounted for using random day and cow intercepts. Causal diagrams were made between algometer measurement and each covariate [(19); diagrams not shown]. Based on those causal diagrams, it was deemed reasonable to use unconditional models for all covariates (i.e., no important confounders were identified for any of the covariates under investigation).

The logistic mixed models were as presented in Equation 1, where Y_ijk_ was the reaction (or lack of reaction) at the i^th^ measurement of the j^th^ day of the k^th^ cow, which was a function of the covariate (X) through the logit function, and followed a binomial distribution with prevalence P of presentation of avoidance reaction. β_0_ was the intercept and β_1_ the regression coefficient for X. The cow, day, and measurement error terms, were v_0k_, u_0jk_, and e_0ijk_, respectively.

The linear mixed models were as presented in Equation 2, where MNT_ijk_ was the MNT (in kg; mean μ and variance σ) for the i^th^ measurement of the j^th^ day from the k^th^ cow. β_0_ was the intercept and β_1_ was the regression coefficient for the covariate (X). v_0k_, u_0jk_, and e_0ijk_ were the cow, day, and measurement error terms, respectively, all assumed to follow an approximately normal distribution.

Again, for the latter model, the fit of different covariance structures (compound symmetry, autoregressive, autoregressive moving average, Toeplitz, and heterogeneous variance compound symmetry) was compared using the Akaike Information Criterion. The compound symmetry correlation structure (equivalent to a conventional random intercept) was shown to provide a fit similar to that of more complex structures and was, therefore, chosen.

Estimates of variances were obtained using a model without predictors. Then, as described by Dohoo et al. (19), we used those estimates to calculate the proportion of the variation in the MNT that was explained by the characteristics of the observation, day, or cow.

Descriptive statistics were performed using SAS version 9.4 (SAS Institute Inc., Cary, NY). Statistical analyses regarding algometer reliability were performed with the same software. All mixed effect models were fitted using MlWin 2.3 (Rasbash, London, UK).

### Sample size calculation

Regarding reliability, we estimated that a sample size of 175 observations (35 cows observed once a day for 5 days) would be sufficient to detect a difference in Kappa values between 0.60 and 0.40, with an alpha of 0.05, a power of 80% and assuming that 90% of cows would respond to pressure with an algometer (20).

Regarding the association between covariates and algometer measurement, we estimated that with a confidence level and a power of 95% and 80%, respectively, a sample of 28 cows, and assuming a standard deviation of 0.68 kg in MNT Fitzpatrick (25), we would be able to detect a difference of at least 0.77 kg between covariate levels.

## Results

The total number of cows enrolled in the project was 36, and five cows had some missing observations. From the total source population of 37 cows, one was excluded from the sampled population due to aggressiveness. Twenty six percent were first parity cows (9/36), 23% were second parity cows (8/36) and the majority, 51% (18/36), were third parity or greater. Furthermore, 28% (10/36) were in early lactation, 31% (11/36) in mid and 42% (15/36) were in late lactation. Mean (± SD) milk production at afternoon milking was 14.0 (± 3.6) L.

### Reliability

**Figure 2:**
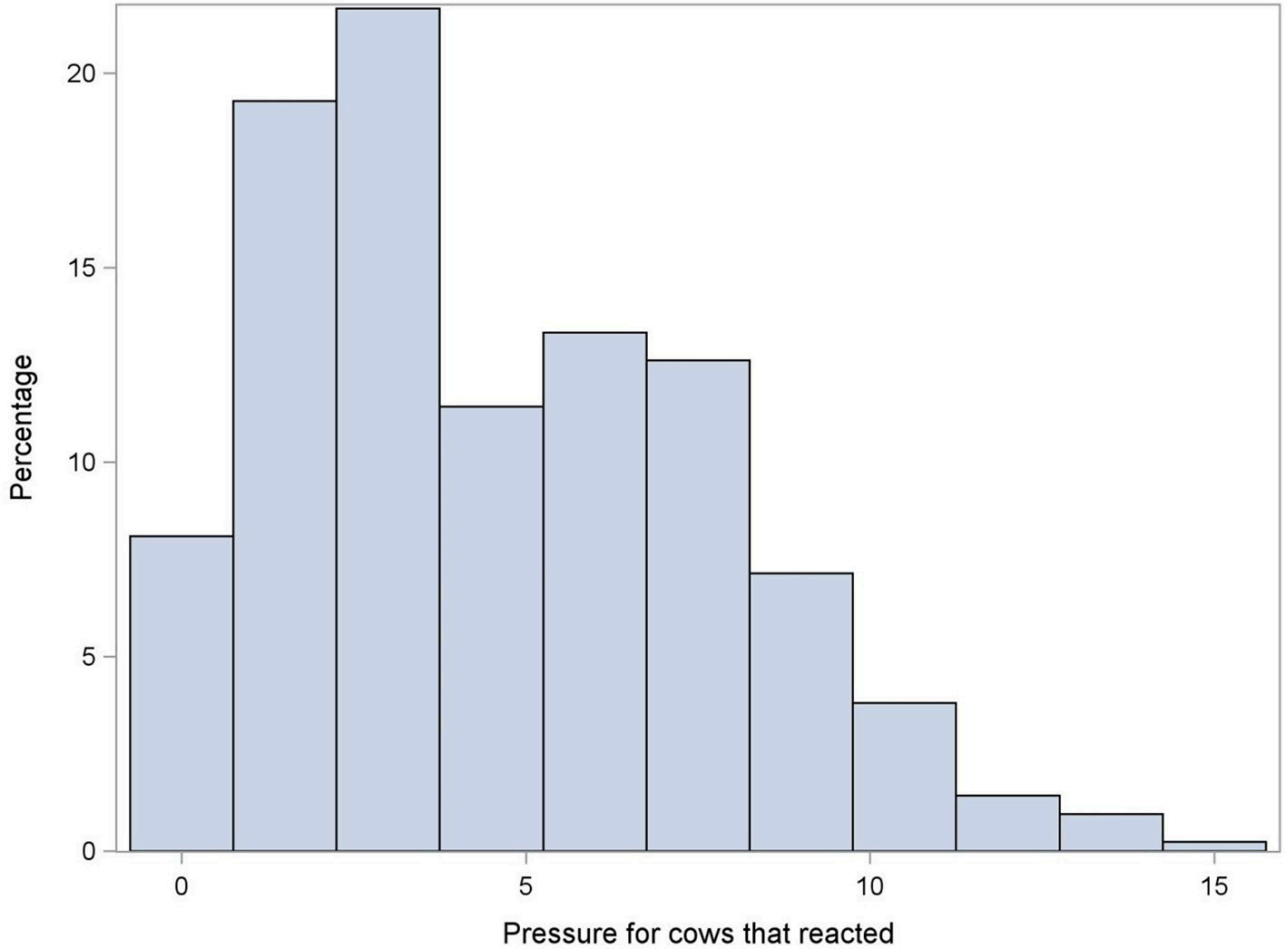
Distribution of the mechanical nociceptive threshold (in kg) measured using a handheld pressure algometer. Data obtained using one measure per day for five consecutive days on 36 milking cows.

**Figure 3:**
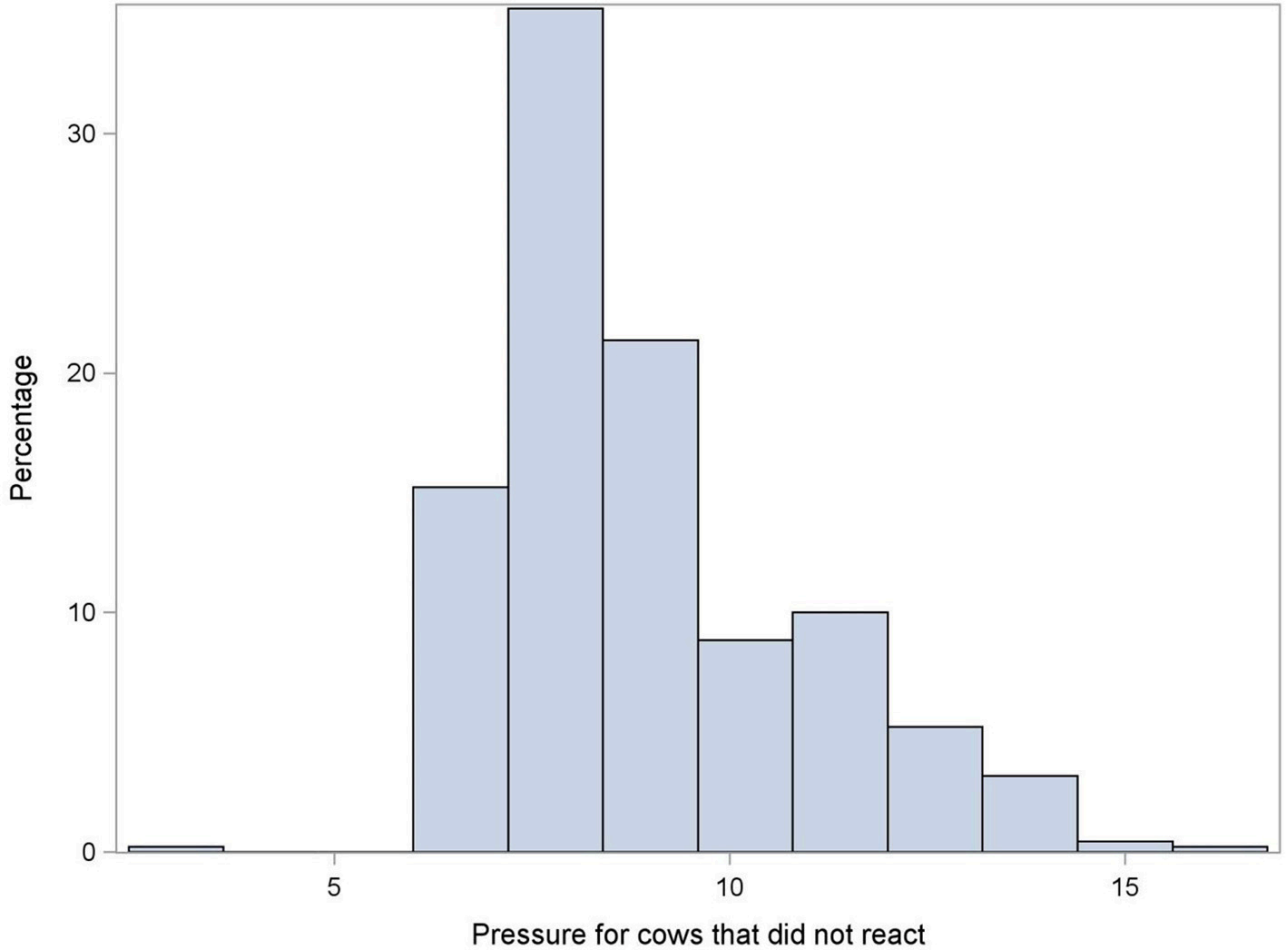
Distribution of the amount of pressure applied using a handheld pressure algometer in cases where cows did not react (in kg; maximum pressure). Data obtained using one measure per day for five consecutive days on 36 milking cows.

Three hundred and forty three pairs of observations were available for the test-retest reliability (36 cows × 5 days × 2 raters, minus 17 pairs of missing observations). An avoidance reaction was observed in half of the 1st measurement observations (49%, 169/343) and in 45% (156/343) of 2nd measurements. Figures 2, 3 show, respectively, the pressure applied in case of reaction (i.e., MNT) and in case of no-reaction (i.e., maximum pressure). Moderate test-retest agreement was observed (Kappa = 0.53; 95% CI: 0.45, 0.63) for the reaction or no reaction to the pressure algometer.

Three hundred and thirty six pairs of observations were available for inter-rater reliability, (36 cows × 5 days × 2 observations, minus 24 pairs of missing observations). An avoidance reaction was observed in 48% (160/336) of rater one and 48% (160/336) of rater two observations. Again, moderate test-retest agreement was observed (Kappa = 0.42; 95% CI: 0.45, 0.63) for the reaction or no reaction to the pressure algometer.

**Figure 4:**
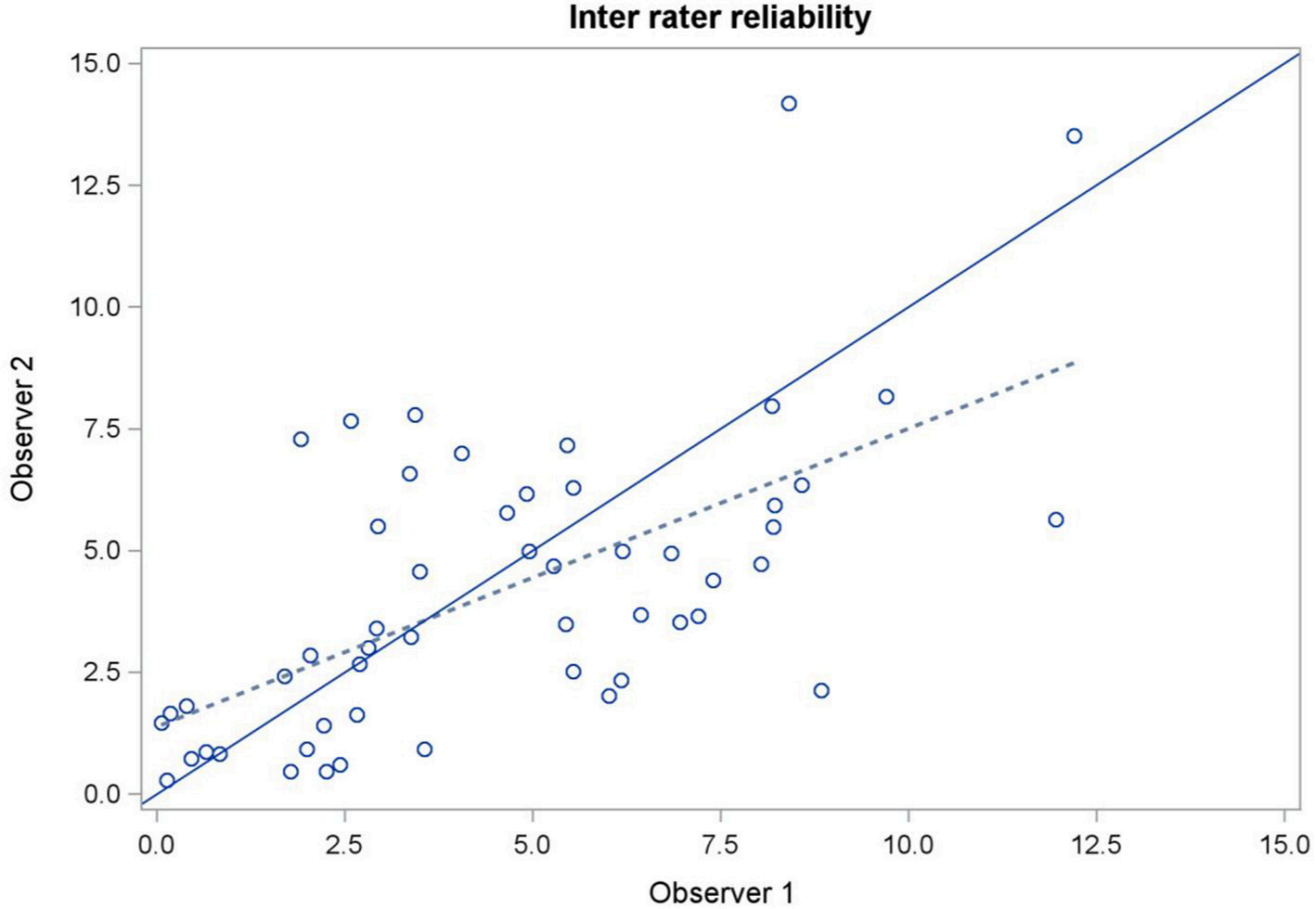
Concordance correlation plot comparing inter-rater reliability for mechanical nociceptive threshold quantified using a handheld pressure algometer. Data obtained using one measure per day for five consecutive days on 36 milking cows. The full line represents the line of perfect concordance and dashed line represents reduced major axis.

**Figure 5:**
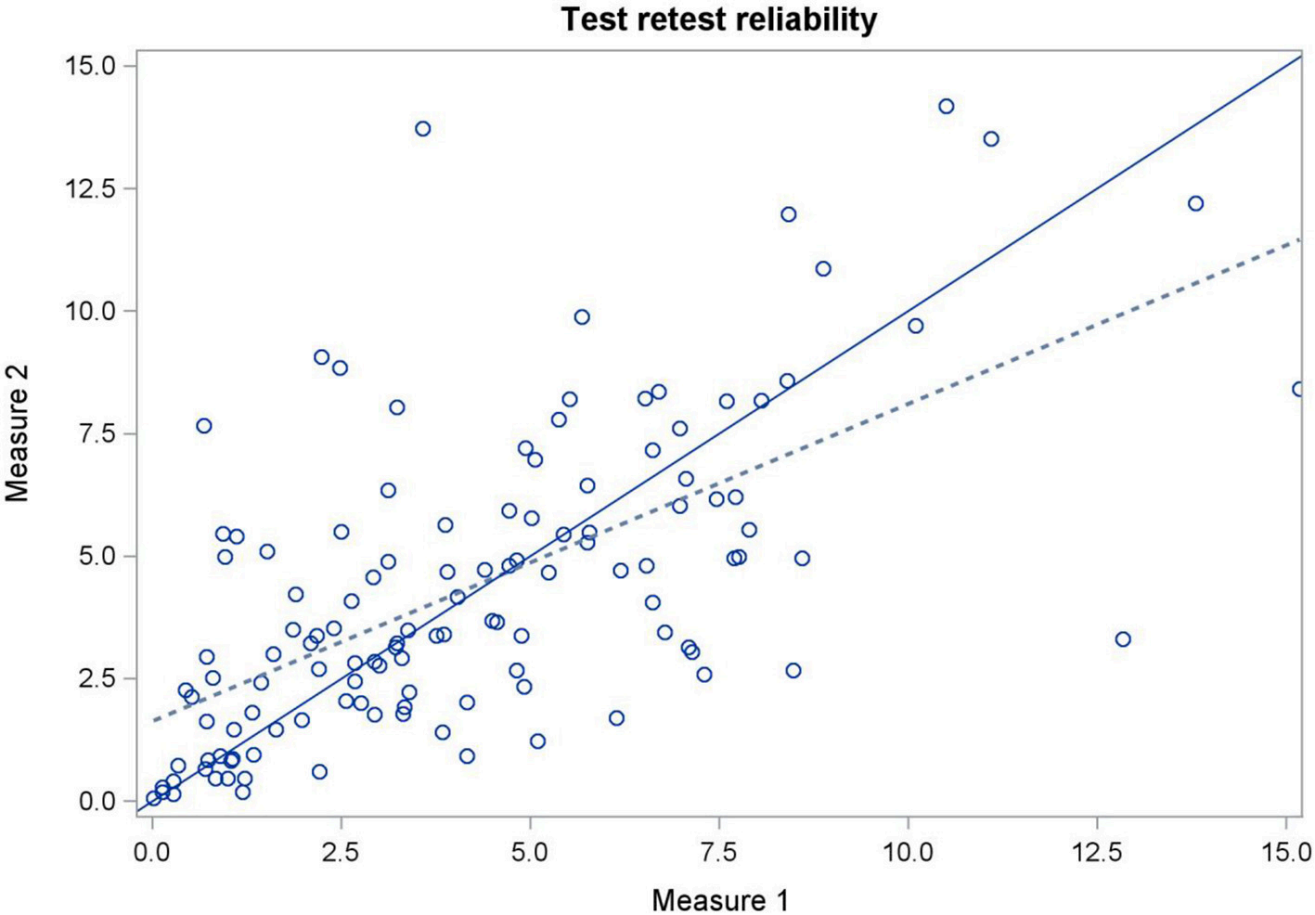
Concordance correlation plot comparing test-retest reliability for mechanical nociceptive threshold quantified using an algometer. Data obtained using two consecutive measures per day for two raters and for five consecutive days on 36 milking cows. The full line represents the line of perfect concordance and dashed line represents reduced major axis.

Regarding the MNT (only in cows reacting to algometer), limits of agreement plots were inspected both for the test-retest and inter-rater analyses and no discernible pattern of disagreement was observed (data not shown). The mean difference between the MNT evaluated in two measurements from the same rater (test-retest reliability) was −0.56 kg (95% CI: −5.40, 4.29). The mean difference between the MNT evaluated by both raters (comparison between the first measurement of both raters; and comparison between the second measurement of both raters) was 0.81 kg (95% CI: −4.44, 6.06). Figures 4, 5 show the CCC plots for inter-rater and test-retest reliability, respectively. Only a small shift of the slopes and, consequently, of the intercepts were observed. Moderate test-retest agreement (CCC = 0.58; 95% CI: 0.46, 0.68) and inter-rater agreement (CCC = 0.54; 95% CI: 0.27, 0.73) were observed.

To further detail test-retest reliability and inter-rater reliability, the effect of raters (A or B) and of order of measurements (1st, 2nd, 3rd, or 4th) on both the qualitative binary measure (reaction vs. no reaction) and on the MNT were investigated. There was a total of 686 observations available for analyses (36 cows × 5 days × 2 raters × 2 observations, minus 34 pairs of missing observations). Raters (*P* = 0.79) and sampling order (*P* = 0.66) did not affect odds of reacting to pressure with an algometer. On the other hand, both rater (*P* < 0.01) and sampling order (*P* = 0.03) affected the MNT (among the 325 cow-observations that reacted). One rater recorded MNT that were on average 0.63 kg (95% CI: 0.17, 1.10) higher than MNT recorded by the other rater. After adjustment for multiple comparisons using the Tuckey-Kramer adjustment, the effect of order of measurements on MNT was only significant between 2nd and 3rd measurements (*P* = 0.03; mean difference 0.92, 95% CI 0.06 to 1.78) but not for any of the other comparisons (i.e., 1st vs. 2nd vs. 3rd vs. 4th). Mean (non-adjusted 95% CI) MNT was 5.25 (4.48, 6.02), 5.35 (4.56, 6.13), 4.43 (3.65, 5.21), and 4.92 (4.12, 5.71) kg for the 1st, 2nd, 3rd, and 4th measurements, respectively. Note that, as per study design, rater A always collected his two measurements (1st and 2nd) prior to rater B's measurements (3rd and 4th).

### Relationship between algometer measurements and covariates

Parity was associated to odds of reacting to pressure with the algometer (*P* = 0.02). Primiparous had equal odds of reacting compared to 2nd parity cows (95% CI for the OR: 0.74, 6.0; *P* = 0.20) and higher odds of reacting than ≥ 3rd parity cows (OR: 3.5; 95% CI: 1.5, 8.2; *P* < 0.01). Unconditional associations between covariates and probability of presenting an avoidance reaction are shown in Table 1.

**Table 1:** Unconditional associations between predictors and odds of reacting to an algometer (reaction vs. no reaction) and between predictors and mechanical nociceptive threshold (in kg; for cows reacting to pressure exerted with an algometer).

		**Reaction vs. no reaction (logistic mixed regression)**				**Mechanical nociceptive threshold (in kg; linear mixed regression)**			
**Parameter**	**Level**	**β**	**SE**	**95% CI**	**Joint *P*-value**	**β**	**SE**	**95% CI**	**Joint *P*-value**
**MODEL 1**									
Intercept		−0.03	0.23			5.45	0.42		
Time after morning milking	0 h	Ref.	Ref.	Ref.	0.92	Ref.	Ref.	Ref.	< 0.01
	4:00 h	−0.02	0.22	−0.4, 0.4		−0.62	0.38	−1.4, 0.1	
	5:30 h	−0.11	0.22	−0.5, 0.3		0.17	0.39	−0.6, 0.9	
	7:30 h	0.10	0.22	−0.3, 0.5		−1.05	0.37	−1.8, −0.3	
	9:00 h	0.02	0.22	−0.4, 0.4		0.08	0.38	−0.7, 0.8	
**MODEL 2**									
Intercept		0.22	0.47			6.17	0.95		
Milk production^a^		−0.02	0.03	−0.1, 0.0	0.56	−0.07	0.06	−0.2, 0.1	0.25
**MODEL 3**									
Intercept		−0.12	0.33			4.92	0.62		
Days in Milk	≤ 100	Ref.	Ref.	Ref.	0.79	Ref.	Ref.	Ref.	0.88
	101–199	−0.03	0.42	−0.8, 0.8		0.39	0.77	−1.1, 1.9	
	≥ 200	0.22	0.43	−0.6, 1.1		0.23	0.80	−1.3, 1.8	
**MODEL 4**									
Intercept		0.75	0.36			3.52	0.56		
Parity	1	Ref.	Ref.	Ref.	0.02	Ref.	Ref.	Ref.	< 0.01
	2	−0.75	0.53	−1.8, 0.3		1.74	0.84	0.1, 3.4	
	≥ 3	−1.24	0.44	−2.1, −0.4		2.47	0.71	1.1, 3.9	

Data generated from an observational study conducted on 36 dairy cows from a teaching farm. Estimates were obtained using logistic (n = 860 observations) and linear (n = 420 observations) mixed regression models accounting for clustering by day and by cow.

a *Milk production in kg per milking*.

β, Regression model coefficient estimate;

SE, Standard error of the mean;

CI, Confidence interval;

*Ref, Reference level*.

The variation in MNT was 59% due to characteristics of the measurement (e.g., time of the day and/or level of activity of the cow at that moment), 10% due to characteristics of the day (e.g., Monday vs. Tuesday), and 31% due to cow characteristics (e.g., production level, age).

Mechanical nociceptive threshold varied with time after milking (*P* < 0.01). The mean (95% CI) MNT, in kg, was 5.45 (4.6, 6.3) immediately after milking, 4.83 (4.0, 5.7) at + 4:00 h, 5.62 (4.8, 6.5) at + 5:30 h, 4.40 (3.6, 5.2) at + 7:30 h, and 5.53 (4.7, 6.4) at + 9:00 h post-milking (Table 1). Therefore, MNT did not increase or decrease constantly during the day. Milk production and DIM were not associated to MNT, but parity was (*P* < 0.01). Compared to primiparous cows, cows in 2nd and ≥ 3 parity had an increment of 1.7 (0.1, 3.4) and 2.5 (1.1, 3.9) on the MNT, respectively (Table 1).

## Discussion

In large animal studies, algometers have been used to assess sensitivity, but their use in assessing udder sensitivity of dairy cows is relatively recent (8) and has never been validated. This study describes, for the first time, the reliability of using a pressure algometer to assess MNT when applied to the udder. The instrument is simple to apply, moderately reliable, and the MNT appears to be affected by many factors.

The number of reactions and no reaction to the algometer was approximately the same within and between raters. However, in previous studies (8) reporting using an algometer on mastitic cows, the lack of reaction to the pressure exerted with the algometer was not mentioned. It was therefore unclear if the use of the algometer led to a reaction in all cows (regardless of evaluation in an infected or uninfected quarter), if the maximum pressure applied was recorded as MNT in cows not showing a reaction, or if cows that did not react were not used in the analyses. In the current study, the high proportion of observations with no reaction may have been caused by the fact that milking cows are accustomed to being handled, touched and milked by humans, and if all cows showed avoidance each time they were milked, the milking process would be difficult for both the cow and the person milking. Other reason could be a very low udder sensitivity, or the development of some degree of tolerance due to repeated sampling. However, other studies in humans have showed that repeated stimulus can also provoke progressive intensification of the perceived pain (21). In future studies, modifying the area of skin contact could be investigated. The use of different types of probe could also be investigated. In the current study, we fixed a 24.2 cm^2^ concave probe head on the pressure point of the algometer to assure a good adaptation to the udder anatomy, and to avoid discomfort due to algometer surface. We could hypothesize that this type of probe does not create a level of discomfort that is sufficient to elicit a response from the majority of cows. A narrower, pencil-shaped, but smooth probe, for instance, could have elicited a higher proportion of response among cows.

Within the same rater, the second measurement lead to slightly lower, but not significant, odds of reaction, which suggests that some cows might have only reacted due to the initial stimulation, therefore gaining some tolerance at the 2nd measurement. Thus, there was some bias due to test repetition (22). If, in the future, the device is used by other researchers, perhaps only 2nd measurements should be used. Moreover, Raundal et al. (23) suggested that a period of habituation to handheld devices used to evaluate MNT improves reliability.

The moderate test-retest and inter-rater reliability, both in Kappa and CCC, showed that the measurement is still somehow reliable. Our results showed that odds of reaction were not influenced by rater nor order of measurements. On the other hand, the quantitative outcome of the pressure algometer stimulation was associated to both factors. There may be some subjectivity for the rater to decide when pressure has to be stopped for reading (i.e., to judge when the cow initiated a reaction). Moreover, differences between raters may also have been caused by difference in promptness of response (i.e., withdrawal of the handheld algometer) once the cow's reaction is noticed. Nevertheless, for research purposes, the use of a single observer would possibly be preferred. In our study, the MNT was also significantly influenced by whether it was the 1st, 2nd, 3rd, or 4th consecutive measurement, but not in a linear way. Since all 1st and 2nd measurements were collected by rater A and all 3rd and 4th were collected by rater B, the rater effect could not be dissociated from the order effect. Nevertheless, cows may have become sensitized by the repeated stimuli and, thus, started to respond more promptly to the pressure applied on 3rd and 4th measurements.

In the current study, raters were not blinded to their own results, nor to the other rater's results, and operator induced variation is a known challenge of handheld tools (23). When designing the current study, we were not aware of the possibility of some cows (51% in our case) not reacting, thus blinding was not considered in the current study. The results obtained, however, suggest that the algometer measurement is not as objective as initially hypothesized. As suggested before, further studies involving different shape of probes may help reducing the proportion of cows not responding to the stimuli. Regarding blinding, we can hypothesize that the absence of blinding in the current study may have led to either no bias or to bias leading to more similar measurements between raters. Thus, the repeatability measure obtained should be considered as a “best case scenario.” As a result, we suggest the inclusion of blinding procedures in future studies.

Within all the covariates tested in the unconditional analysis, the only one that influenced the qualitative outcome was parity of the cow, with cows from lower parities having higher odds of reacting than older cows. Analogous results were obtained for the quantitative analysis, since primiparous cows had lower MNT compared to multiparous. The higher chances of reaction along with the lower MNT observed in primiparous cows might be a result of their lower experience in being milked, handled or touched in the udder area compared to multiparous cows. If this hypothesis is correct, then algometer results would be an indicator of the cow's experience, instead of an indication of increased sensitivity due to udder distension. Another possibility is that the lower MNT in primiparous were caused by less quantity of secretory tissue (24) and smaller udder than multiparous cows. In such a case, algometer results would actually be a measure of cow increased sensitivity due to udder distension. These results are in agreement with those of Fitzpatrick (25), who found that multiparous cows tolerated on average 0.77 kg more pressure than primiparous cows. Future studies could help confirming these differences and clarifying the reasons behind those.

Although time after milking did not affect the qualitative algometer outcome, it did affect the MNT. However, MNT was not proportional to udder repletion (i.e., number of hours post-milking). It is hypothesized that algometer result was modulated by cows' activity when the measurement was taken (i.e., if they were lying down vs. up and eating at the moment of sampling), similarly to what was found previously in piglets (14). In the current study, the interval between the morning and afternoon milking (time of sampling process) was of ~9 h. However, according to Ayadi et al. (26), even though cisternal and alveolar milk volume increased proportionally to milking interval, cisternal and alveolar repletion plateau were only reached 20 h and 16 h after milking, respectively. Thus, it is possible that the study duration (i.e., 9 h) was not sufficient to lead to the accumulation of a volume milk that would cause an increased udder sensitivity. Future studies evaluating the effect of time since milking, but using longer milking interval, would possibly lead to different conclusions. Algometer measurements following abrupt cessation of milking at drying-off (1) or following incomplete milking at the beginning of the lactation (3, 4), could also possibly lead to different conclusions.

According to Caja et al. (27), cisternal milk volume decreased (by 49%) between early and mid-lactation, while alveolar milk volume decreased mostly between mid and late lactation (by 68%). So if MNT is related to milk volume in the udder, we would expect to have cows in early lactation reacting faster than cows in late lactation. In the current study, in the same way that high-producing cows did not show lower MNT to the pressure algometer, differences between stages of lactation were not observed.

In conclusion, the algometer had moderate test-retest and inter-rater reliability on both qualitative (reaction vs. no reaction) and quantitative outcomes (i.e., MNT). Cow MNT was influenced by various extraneous covariates, including time of the day at which the measurement was taken, and cow characteristics such as parity. These results suggest that these factors should be taken into account when using an algometer to measure MNT on the udder of dairy cows. Algometer results seem to be highly variable and may actually measure concepts that are quite different than udder sensitivity. Algometer usage in research setting to quantify sensitivity when applied to the udder of dairy cows should, therefore, be considered cautiously or this methodology should be further developed. At the very least, if using these devices, an attempt to match animal studied (e.g., exclusion, pairing, conditional models) on time of the day and cow characteristics should be made. In future research, understanding what influences the changes in MNT during the day (e.g., the cow, the type of activity such as lying down or eating, the time since last milking, diurnal cycles, etc.) could help standardizing the algometer MNT measurement.

## Author contributions

CK: conception or design of the work, data collection, data analysis and interpretation, drafting the article, final approval of the version to be published. TD: critical revision of the article, final approval of the version to be published. J-PR, JD, and SD: conception or design of the work, data analysis and interpretation, critical revision of the article, final approval of the version to be published.

### Conflict of interest statement

The authors declare that the research was conducted in the absence of any commercial or financial relationships that could be construed as a potential conflict of interest.
